# Object Shape Recognition Using Sparse Soft Capacitive Tactile Sensors for Robotic Hands

**DOI:** 10.3390/s26175583

**Published:** 2026-09-02

**Authors:** Xinmeng Ding, Yuting Zhu, Mengdi Chen, Wee Chen Gan, Shaohua Wang, Kean Aw

**Affiliations:** 1 Department of Mechanical and Mechatronics Engineering, University of Auckland, Auckland 1010, New Zealand; xdin247@aucklanduni.ac.nz (X.D.); swan932@aucklanduni.ac.nz (S.W.); 2 School of Engineering and Built Environment, Griffith University, Nathan, QLD 4111, Australia; y.zhu2@griffith.edu.au; 3 College of Textiles, Donghua University, Shanghai 201620, China; 1225041@mail.dhu.edu.cn; 4 School of Energy and Chemical Engineering, Xiamen University Malaysia, Sepang 43900, Malaysia

**Keywords:** sparse tactile sensing, robotic hand perception, object shape recognition, multimodal tactile sensing

## Abstract

Reliable tactile object shape recognition on robotic hands is often achieved using dense sensor arrays or vision-based tactile skins, which increase fabrication complexity and computational cost. This work demonstrates that high-recognition performance can instead be achieved through principled sparse sensing. A minimal multimodal tactile system is developed by fusing soft capacitive stretch sensors at the proximal interphalangeal and metacarpophalangeal joints of the fingers with a sparse six-element palmar pressure array, integrated into a human-like hand mechanically constrained to emulate robotic grasping under a controlled and repeatable protocol. Using an ANOVA-based channel selection, low-informative metacarpophalangeal signals are identified and removed, reducing the number of sensors at the finger joints while improving classification accuracy. A lightweight multi-layer perceptron operating on this low-dimensional input achieves 95.4% size-invariant recognition accuracy across 12 rigid objects representing four geometric primitives—cuboid, sphere, cylinder, and cone—outperforming the denser baseline. Ablation studies confirm the complementary roles of finger-joint deformation, which encodes curvature cues, and palmar force distribution, which captures contact topology; neither modality alone achieves comparable performance. Beyond accuracy, the proposed design reduces sensor count, wiring, and computational requirements, enabling embedded-ready deployment. The results show that data-driven sensor placement, rather than sensors at all finger joints, can yield sufficient grasp-based shape recognition, offering practical guidance for tactile perception in resource-constrained robotic hands.

## 1. Introduction

Robotic hands rely heavily on tactile information to interact with the physical world. When vision is unreliable due to occlusion, clutter, poor lighting, or visually similar objects, touch becomes essential for recognizing object shapes and guiding manipulation [1,2,3]. Humans excel at this ability, identifying objects through touch by combining information from finger posture, joint motion, and contact forces across the hand; bringing comparable capability to robotic hands remains a long-standing challenge in robotics [1,3,4].

Over the past decade, advances in soft robotics, flexible sensors, and machine learning have enabled promising tactile recognition systems [4,5,6,7,8,9]. High-resolution tactile arrays, vision-based tactile sensors [6], and multimodal sensing skins have demonstrated impressive performance in controlled settings, but they often depend on dense layouts, complex wiring, specialized fabrication, and significant computation, factors that make real-time deployment on embedded, mobile, or resource-constrained robotic platforms difficult [8,9,10,11,12,13,14,15,16]. These practical limitations motivate a shift from “more sensors” to better-placed sensors that capture the most informative tactile cues with minimal hardware.

In human grasping, shape is not inferred solely from detailed pressure maps. It emerges from the relationship between finger bending and the distribution of forces across the palm: joint configuration captures overall grasp posture, while contact forces reflect local surface interactions. When combined, these complementary cues can be highly informative even in sparse form. Guided by this observation, we demonstrate that reliable shape recognition can be achieved using a minimal set of strategically placed tactile sensors, rather than dense arrays. Instead of large, multifunctional skins, two simple, complementary modalities are employed: soft capacitive stretch sensors at finger joints to measure joint deformation, and capacitive pressure sensors on the palm to capture contact forces. This modular sensing strategy reduces fabrication and integration complexity while preserving the key tactile cues needed for recognition. Here, a gloved human hand, where the sensors are attached to the glove, is used to mimic a robotic hand.

A central question in sparse tactile sensing is which sensors matter. Sensor placement is often driven by intuition or anatomical analogy, thereby risking the retention of redundant or weakly informative channels. To address this, a data-driven sensor selection method, based on analysis of variance (ANOVA) to quantify the contribution of each tactile channel to object discrimination [7], was introduced. The analysis shows that metacarpophalangeal (MCP) joint sensors provide little shape-specific information during a power grasp. Removing these channels reduces the configuration from 16 to 11 sensors and, importantly, improves recognition performance rather than degrading it, reflecting the value of principled feature selection in tactile perception [7,10,11,12].

For sensor fusion, a compact multi-layer perceptron (MLP) was adopted. This choice reflects a deliberate focus on simplicity and practicality: our goal is not to maximize accuracy through complex temporal models or massive datasets, but to demonstrate that carefully selected, low-dimensional tactile inputs can support sufficient, size-invariant shape recognition with lightweight models suitable for real-time robotic deployment [2,7,14,17,18,19,20]. However, we acknowledge long-standing challenges in tactile sensing, i.e., drift, hysteresis, limited dynamic range, environmental sensitivity, and sensor placement, which complicate perception pipelines and motivate parsimonious designs [10,11,12]. Likewise, physics-based pipelines can demand detailed contact parameters and become computationally expensive for real-time control [17,18], and our approach complements these by emphasizing the data-driven fusion of sparse but informative signals.

The approach on four geometric primitives, i.e., cuboid, sphere, cylinder, and cone, is evaluated across multiple size scales using a human-like robotic hand platform mechanically constrained to emulate key aspects of robotic grasping [21,22,23,24]. Extensive experiments and ablations show that the optimized 11-channel configuration achieves 95.4% recognition accuracy and outperforms both denser and single-modality setups, underscoring that dense tactile sensing is not a prerequisite for reliable shape recognition [8,9,10,11,12,15,16,25,26]. Instead, thoughtful sensor placement, simple hardware, and efficient learning can deliver strong performance while reducing system complexity, offering practical guidance for tactile perception in robotic hands, where robustness, scalability, and real-time operation are essential [4,6,7,8,9,15,16,17,18,19,20,25,26]. The objective of this work is to investigate whether a strategically selected sparse sensor configuration can support effective shape recognition within the specific grasp-based tactile recognition framework examined here.

## 2. Concept of Proprioception and Object Shape Recognition with a Robotic Hand

### 2.1. Biomimetic Design and Sensor Placement

A gloved human hand is used as a testbed to mimic a robotic hand, exploiting its morphology and mechanoreceptor distribution to guide sensor placement [21]. Sensors are positioned at the Metacarpophalangeal (MCP) and Proximal Interphalangeal (PIP) joints—key contributors to grasp variability—and on the central palm for force mapping. This biomimetic strategy maximizes information with minimal sensors [22].

### 2.2. Shape Recognition Framework

**Figure 1:**
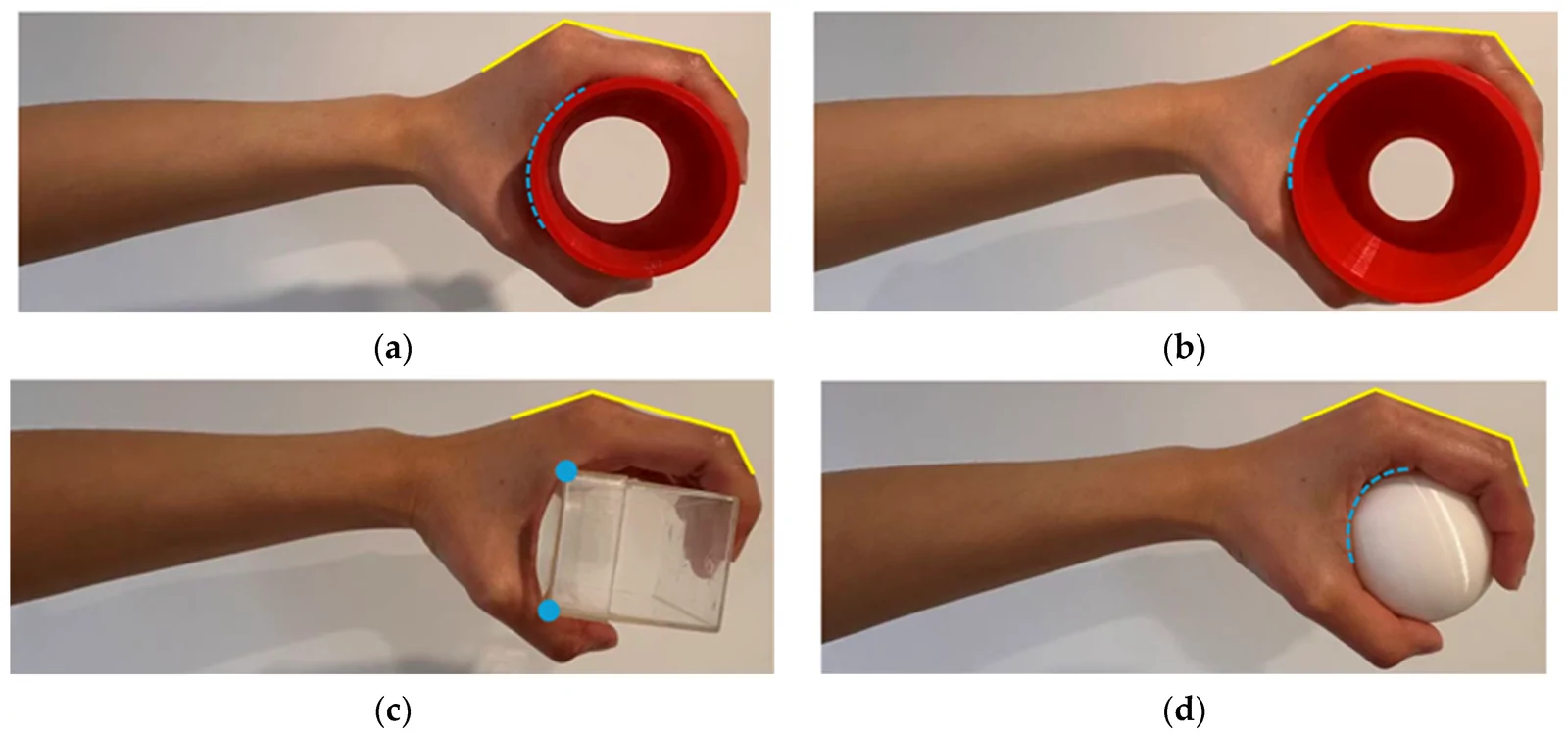
Distinct hand configurations and force distributions for four basic shapes. (**a**) Cylinder—progressive MCP–PIP flexion; elongated longitudinal force with gradual gradients. (**b**) Cone—greater flexion near apex; asymmetric wedge-shaped force concentrated at the narrow end. (**c**) Cuboid—more extended fingers aligned to flat faces; rectangular force with sharp boundaries and emphasis at edges/vertices. (**d**) Sphere—uniform MCP–PIP flexion forming a dome; centralized, circular, radially symmetric force pattern. Legend: Blue regions indicate palmar force distribution; yellow highlights finger flexion.

Object geometry is encoded in two measurable signatures: joint angles (macroscopic posture) and palm force distribution (local contact mechanics). Four basic shapes are considered in this work, i.e., cylinder, cone, cuboid, and sphere, which will produce distinct patterns: Cylinder: smooth MCP–PIP flexion; elongated force gradient. Cone: greater flexion near apex; wedge-shaped force map. Cuboid: extended fingers aligned to flat faces; rectangular force map. Sphere: uniform flexion forming a dome; circular force pattern [23,24]. See Figure 1 and Table 1 for the fingers and force distribution when grasping objects of four different shapes.

### 2.3. Sensor Technology

**Figure 2:**
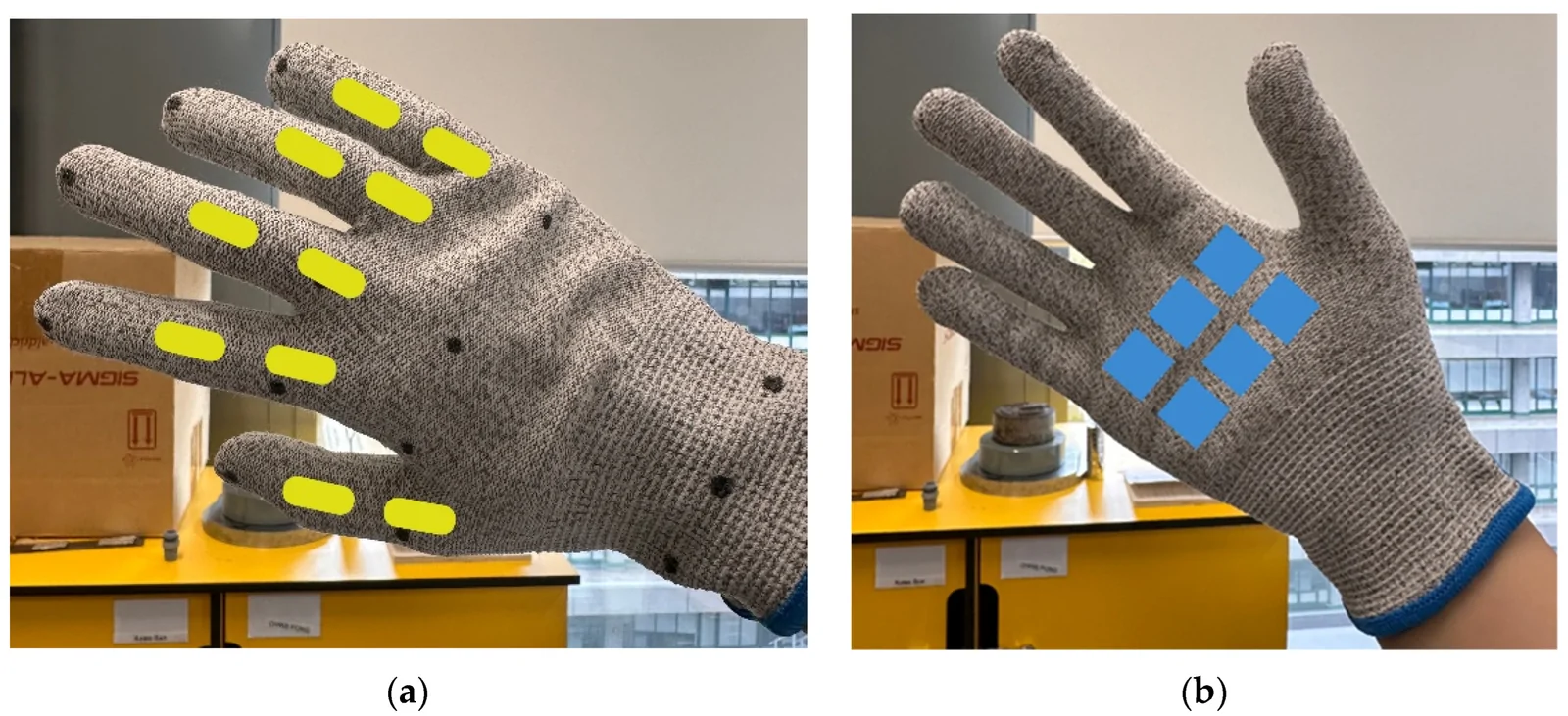
Sensor placement strategy for sparsely placed tactile sensing. (**a**) Joint-angle sensors (yellow) on MCP and PIP joints capture kinematic variability across shapes. (**b**) Palmar pressure sensors (blue) cover the central load-bearing zone to record informative contact maps. Legend: Yellow sensors measure joint flexion (kinematic signatures); blue sensors capture force distribution (tactile signatures).

Capacitive sensors measure geometric changes for joint angles (via electrode overlap/distance) and dielectric compression for force. Benefits include low hysteresis, high repeatability, minimal drift, ultra-low power, and easy integration with microcontrollers. Flexible dielectric layers ensure durability under repeated deformation [25,26]. Figure 2 shows the targeted position of the capacitive sensors.

### 2.4. Contributions and Implications

Strategic sensor placement, biomimetic design, and multimodal fusion, implemented with modern machine learning, enable robust, human-like recognition of fundamental 3D shapes without dense sensor arrays or vision systems [25,27]. This algorithm-centric approach offsets hardware simplicity with processing intelligence, yielding systems that are more capable, practical, and deployable, while clarifying that dense sensing for object recognition can be avoided.

## 3. Flexible Stretch and Pressure Sensors

Two sensor types are developed: stretch sensors positioned at finger joints (PIP and MCP locations) to measure joint angles via capacitance changes during flexion, and pressure sensors distributed across the palm to detect contact force during object grasping. Both sensor configurations employ flexible, low-cost materials (carbon-black/Ecoflex 00-30 composites and compressible sponge dielectrics) that accommodate large mechanical deformations while maintaining stable electrical performance, thereby providing a 16-channel sensing system suitable for object shape recognition.

Two types of capacitive sensors are used, i.e., stretch and pressure sensors. The stretch sensors attached to the finger joints will change their capacitance when bent due to stretching. The pressure sensors will exhibit a change in capacitance when the soft dielectric between the capacitive sensor plates is compressed by the applied force.

### 3.1. Flexible Stretch Sensor

**Figure 3:**
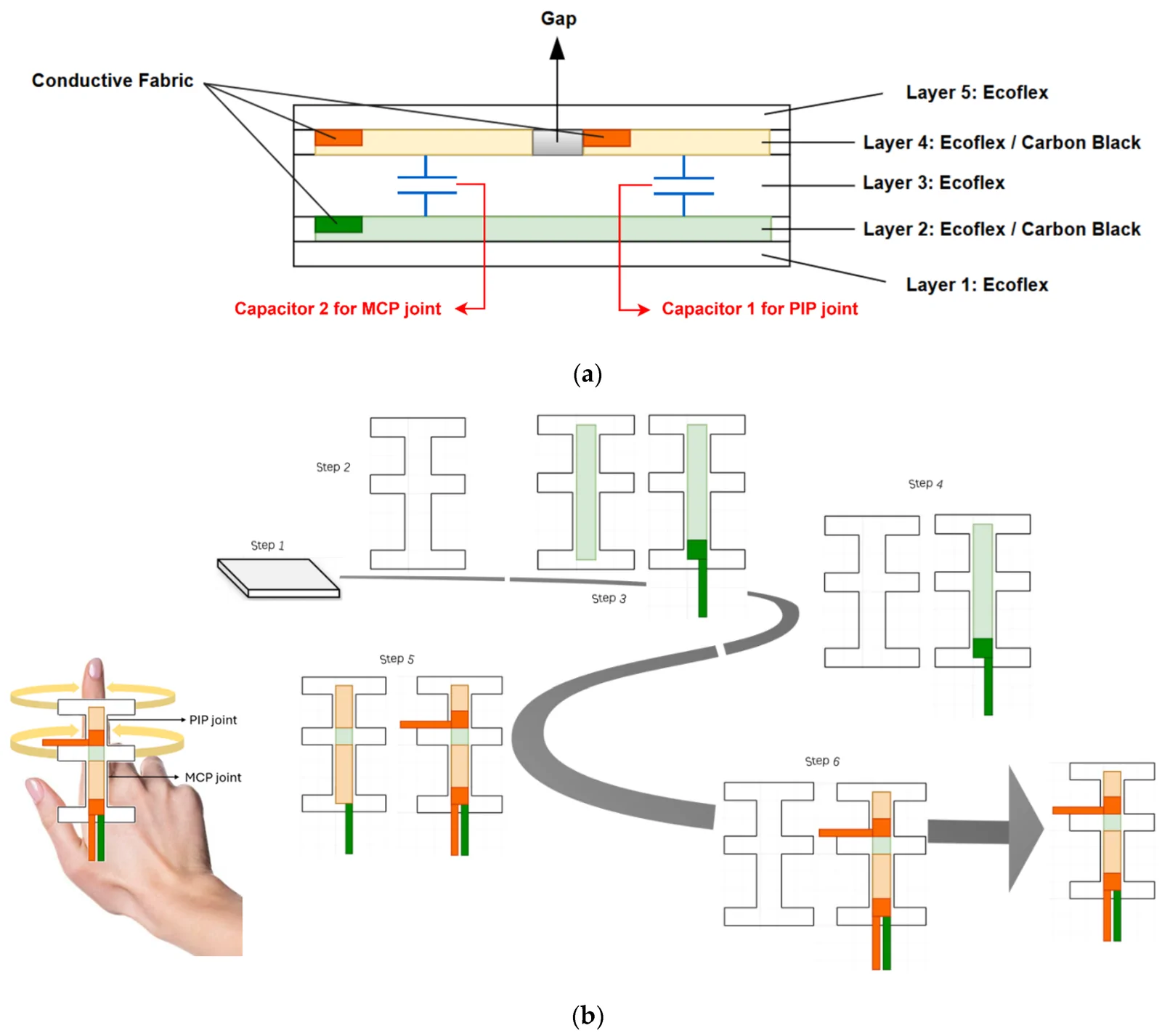
Stretch sensors. (**a**) Cross-sectional schematic of finger joint stretch sensors, (**b**) showing the lateral tabs and the placement on the finger’s PIP and MCP joints.

There are two sensor variants: standard and compact. Stretch sensors are used at the finger joints (PIP and MCP) to measure joint deformation during object grasping by sensing changes in capacitance, exploiting the relationship between mechanical strain and electrode separation distance to transduce joint-angle changes into measurable electrical signals. As the joint angle increases, the stretch sensor elongates, reducing the distance between the two electrodes and increasing the capacitance. The measured capacitance is therefore used to quantify the amount of joint bending. Two sensor size variants accommodate anatomical variation: a standard variant suitable for the index, middle, and ring fingers, featuring 25 mm PIP and 35 mm MCP electrode segments, and a compact variant for the thumb and little finger with 15 mm PIP and 25 mm MCP segments (total conductive length reduced by 20 mm). This size differentiation ensures optimal sensor-to-joint alignment across all five fingers, maximizing strain localization at each target joint while preventing electrode overlapping with adjacent phalanges. The dimensional scaling maintains consistent 10 mm inter-electrode gaps and identical lateral width (10 mm) across both variants, preserving fabrication compatibility and measurement consistency. Figure 3 shows the stretch sensor and where the joint deformation of the PIP and MCP joints can be determined.

Table 2 provides detailed dimensions for the two types of stretch sensors; standard variant for the index, middle and ring fingers and compact variant type for the thumb and little finger. See Supplementary Information for details of the materials and fabrication process for the stretch sensor.

### 3.2. Pressure Sensor

The palm pressure sensor functions as a parallel-plate capacitor with a three-layer sandwich structure: flexible top and bottom electrodes are separated by a soft, elastic material (a sponge). When force is applied, the sponge compresses, bringing the electrodes closer together and increasing the capacitance. An increase in capacitance corresponds to an increase in applied pressure. A total of six pressure sensors arranged in a 2-by-3 array are used as the palm sensor. See Supplementary Information for details of the materials and fabrication process for the pressure sensor.

### 3.3. Sensor Characterization

Both stretch and pressure sensors were subjected to systematic testing protocols to quantify key performance metrics: sensitivity and hysteresis. These characteristics directly impact recognition accuracy by determining how faithfully sensor outputs represent mechanical stimuli during object grasping.

Both stretch and pressure sensors are characterized using a unified Gauge Factor framework. For stretch sensors measuring joint deformation, the conventional strain-based Gauge Factor (GF_ε) is defined as the normalized capacitance change per unit strain: $GF\_\epsilon =\frac{\left(\Delta C/C_{0}\right)}{\epsilon }$. For pressure sensors measuring contact force, an analogous force-based Gauge Factor (GF_F) is defined as the normalized capacitance change per unit applied force: $GF\_F=\frac{\left(\Delta C/C_{0}\right)}{F}$, with units of N^−1^.

#### 3.3.1. Flexible Stretch Sensor Characterization

The initial capacitance values ($C_{0}$) of the sensors were measured and compared with theoretical values calculated from the parallel-plate capacitor model $C=\frac{\varepsilon_{0}\varepsilon_{r}A}{d}$. The measured C_0_ for the Standard Variant’s MCP segment averaged 15 pF (theoretical: 10 pF), and the PIP segment averaged 9 pF (theoretical: 7.4 pF). For the Compact Variant, the MCP segment averaged 14 pF (theoretical: 7.4 pF), and the PIP segment averaged 6 pF (theoretical: 4.9 pF). This variation is due to the dielectric thickness not being uniform across the sensor, which validates the fabrication process and provides a baseline for subsequent sensitivity calculations.

Stretch sensors were mounted in a custom tensile testing apparatus consisting of a manual translation stage, a rigid acrylic fixture, an LCR meter (Keysight E4980AL, Keysight Technologies, Santa Rosa, CA, USA) for capacitance measurement at 25 kHz, and a digital caliper. Each sensor was subjected to incremental tensile strain from 0% to 50% of its original length in 5% steps, with capacitance recorded after a 10 s stabilization period at each strain level. The sensor’s sensitivity was defined as the normalized capacitance change per unit strain, known as the Gauge Factor (GF), calculated using the formula $GF=\frac{\left(\Delta C/C_{0}\right)}{\epsilon }$, where ΔC is the capacitance change, $C_{0}$ is the initial capacitance, and ϵ is the applied strain.

**Figure 4:**
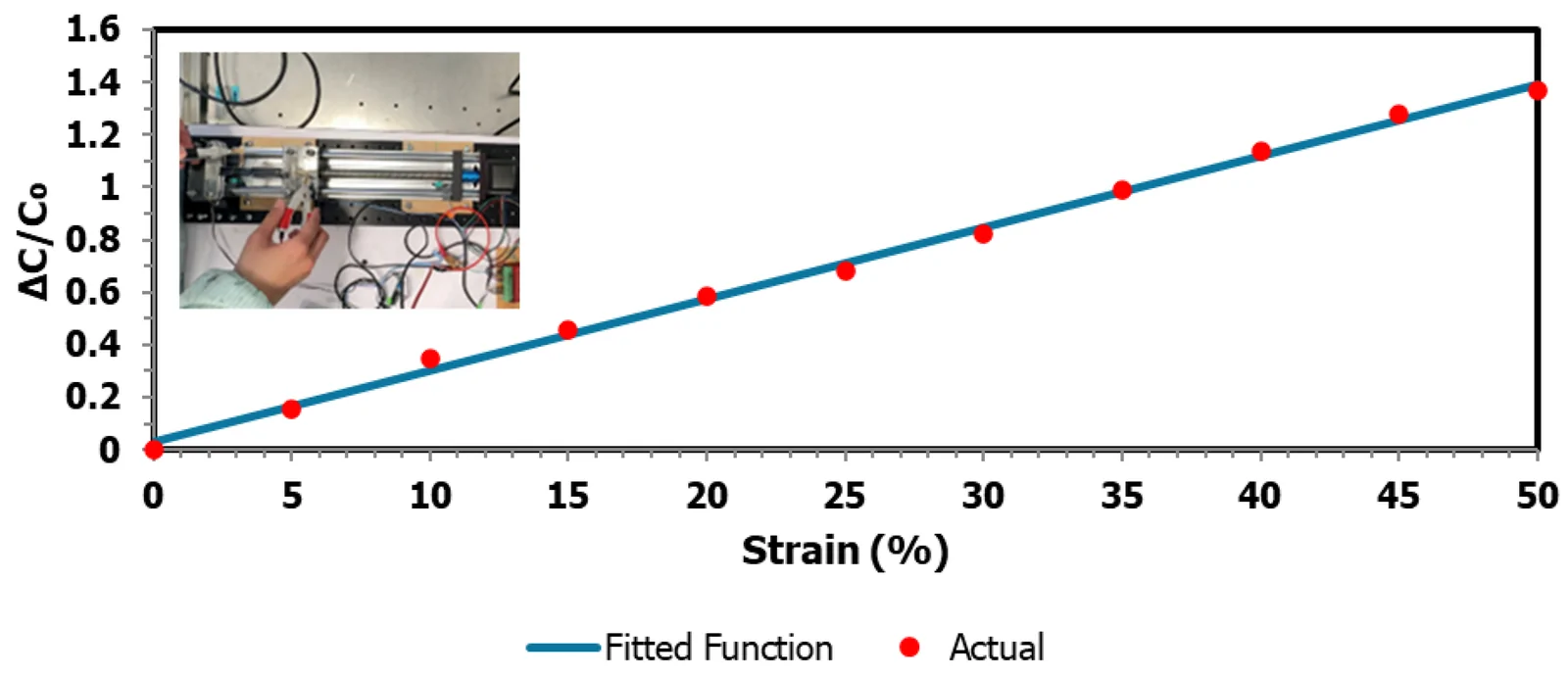
A plot of the capacitance versus strain of a stretch sensor (standard variant, MCP segment, C_0_ = 15 pF) (Insert: a testing rig used to characterize the stretch sensor).

Figure 4 demonstrates the normalized capacitance response (ΔC/C_0_) versus applied strain, revealing excellent linearity (R^2^ = 0.987). The measured GF is approximately 2.67, indicating a 2.67% relative change in capacitance per 1% strain. At the maximum tested strain (50%), the sensor achieves ΔC/C_0_ = 1.373, corresponding to a 137% increase from the initial value, providing a substantial signal magnitude for robust joint angle measurement during finger flexion.

The complete loading–unloading cycle from 0% to 50% strain was performed at a constant speed.

The hysteresis error was calculated as the maximum difference in normalized capacitance (ΔC/C_0_) between the loading and unloading curves at any given strain point, expressed as a percentage of the full-scale output (FSO):

(1) $$\mathrm{Hysteresis}\;\mathrm{Error}=\frac{|(\Delta C/C_{0}{)}_{\mathrm{loading}}-(\Delta C/C_{0}{)}_{\mathrm{unloading}}{|}_{\max}}{(\Delta C/C_{0}{)}_{\max}}\times 100\%$$

**Figure 5:**
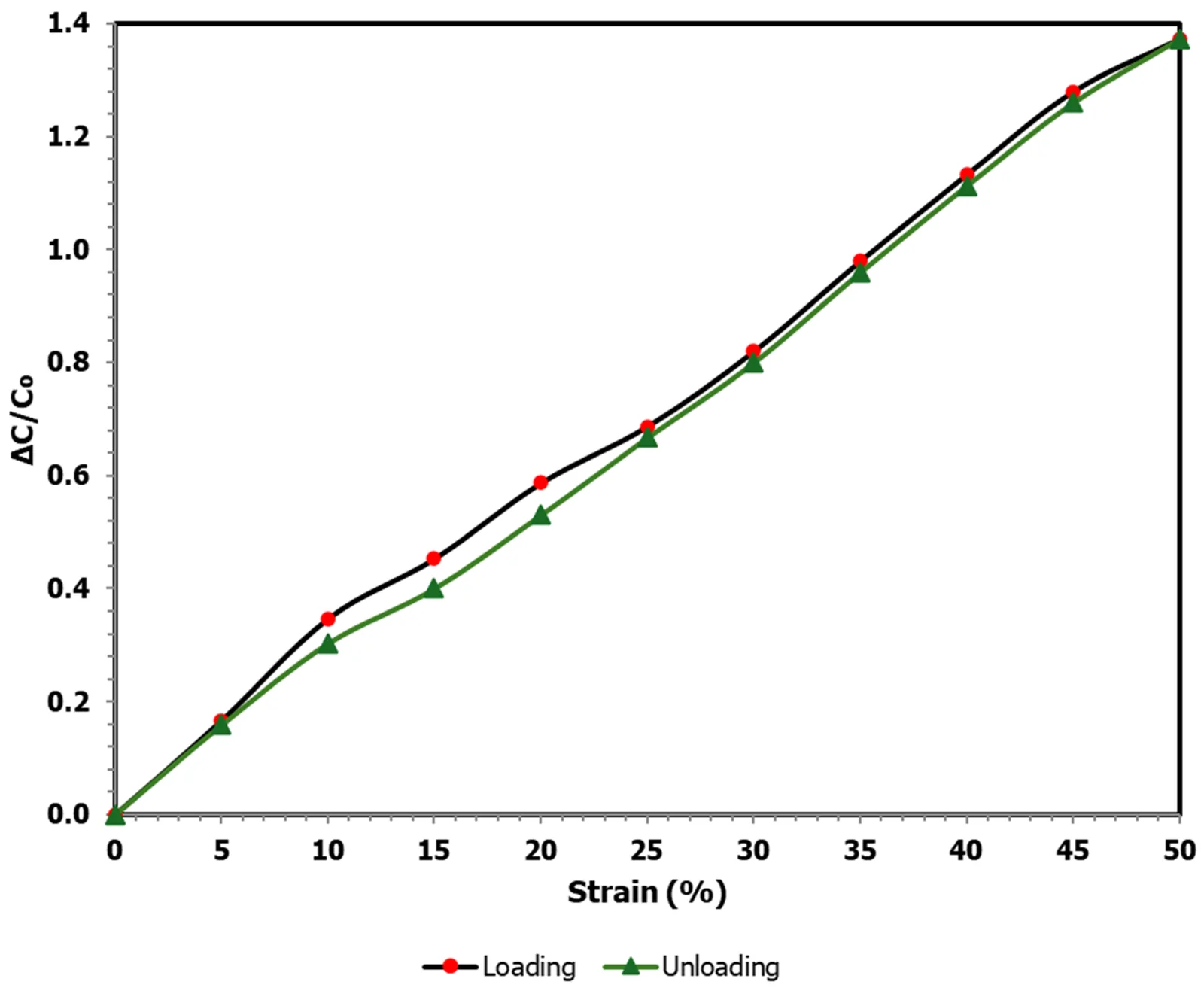
A plot of stretch sensor stretched up to 50% strain and then relaxed, showing the hysteresis.

Figure 5 presents the hysteresis characterization. The maximum hysteresis error of 4.2% FSO occurs at 20% strain, where the difference between loading (ΔC/C_0_ = 0.587) and unloading (ΔC/C_0_ = 0.530) is most pronounced. This hysteresis magnitude is significantly lower than typical resistive strain sensors (which often exhibit 10–15% hysteresis) [28] and validates the advantages of capacitive transduction for flexible sensing applications.

The curves converge at both endpoints (0% and 50% strain), confirming complete elastic recovery and absence of plastic deformation in the Ecoflex matrix. The relatively small hysteresis in the 25–50% strain region (approximately 1.5%) suggests that the sensor material behavior stabilizes at higher deformations, making it particularly suitable for repetitive flexion–extension cycles in robotic hand applications.

#### 3.3.2. Palm Pressure Sensor Characterization

The initial capacitance (C_0_) averages approximately 8 pF per element under no load, consistent with the large initial electrode separation imposed by the thick, compressible sponge dielectric.

Palm pressure sensors were evaluated using a controlled compression protocol. The sensor was positioned on a flat, rigid platform, with standard calibrated weights applied incrementally through a non-conductive, flat-ended cylindrical indenter (0–4 N range, selected based on physiologically relevant contact forces during manual grasping [29]). Capacitance measurements were acquired using a capacitance meter after a 5 s force stabilization period at each loading increment.

**Figure 6:**
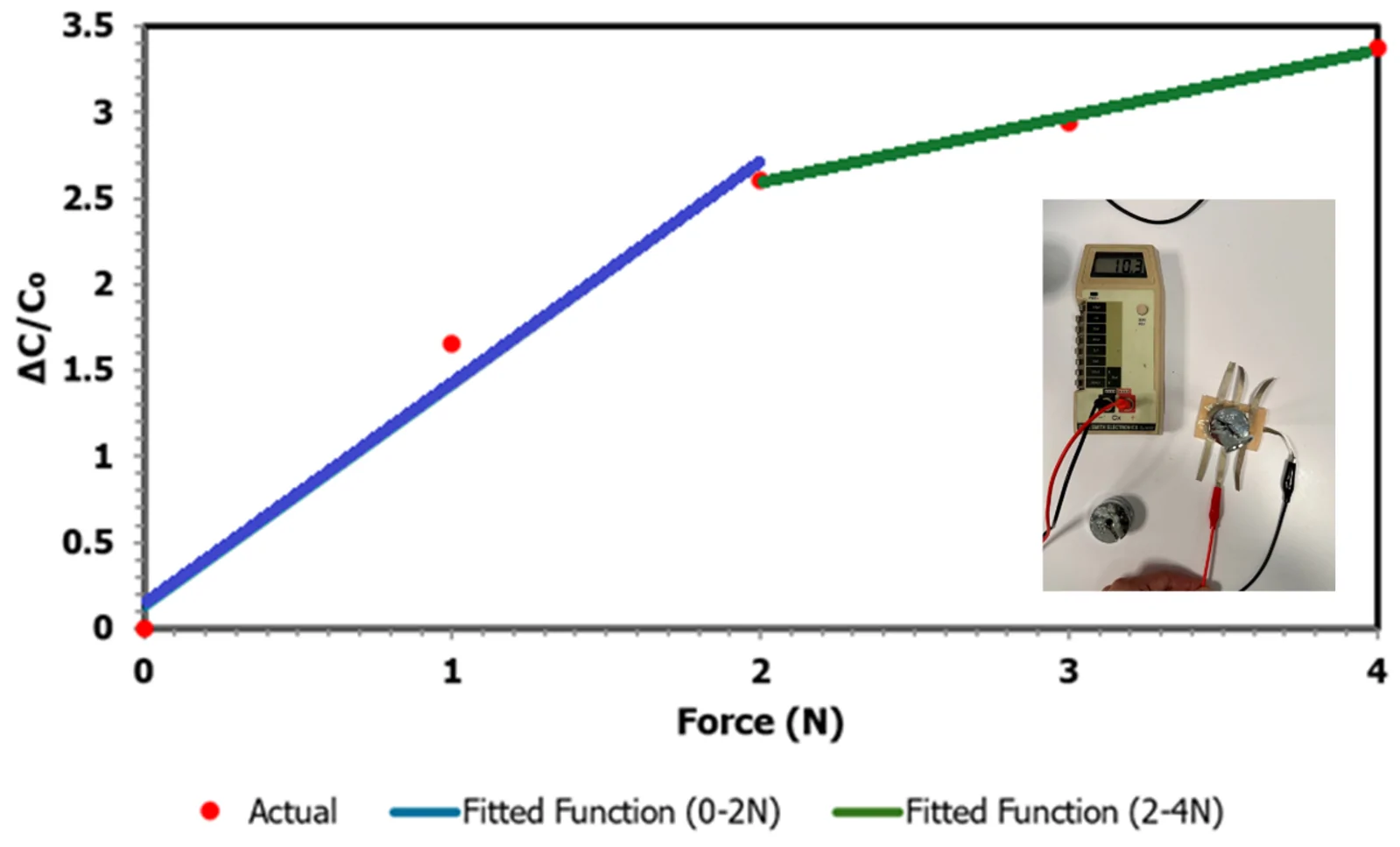
A plot of the capacitance versus applied force of the palm pressure sensor array (averaged across 6 elements, C_0_ ≈ 8 pF). (Insert: a characterization setup for the palm pressure sensor.).

Figure 6 presents the sensitivity characterization showing a distinctive bi-linear response with two clearly defined operational regions. Measured data points (red circles) reveal high sensitivity in the low-force region (0–2 N) with a steep slope of approximately 10 pF/N, transitioning to reduced sensitivity in the high-force region (2–4 N) with a slope of approximately 3.5 pF/N.

Both linear segments exhibit excellent fit (R^2^ > 0.99), demonstrating a predictable, repeatable sensor response across the entire operational range. The sensor achieves a total capacitance change of 27 pF over the 0–4 N range (from 8 pF to 35 pF), providing a dynamic range of 4.4:1 suitable for discriminating contact forces during object grasping.

**Figure 7:**
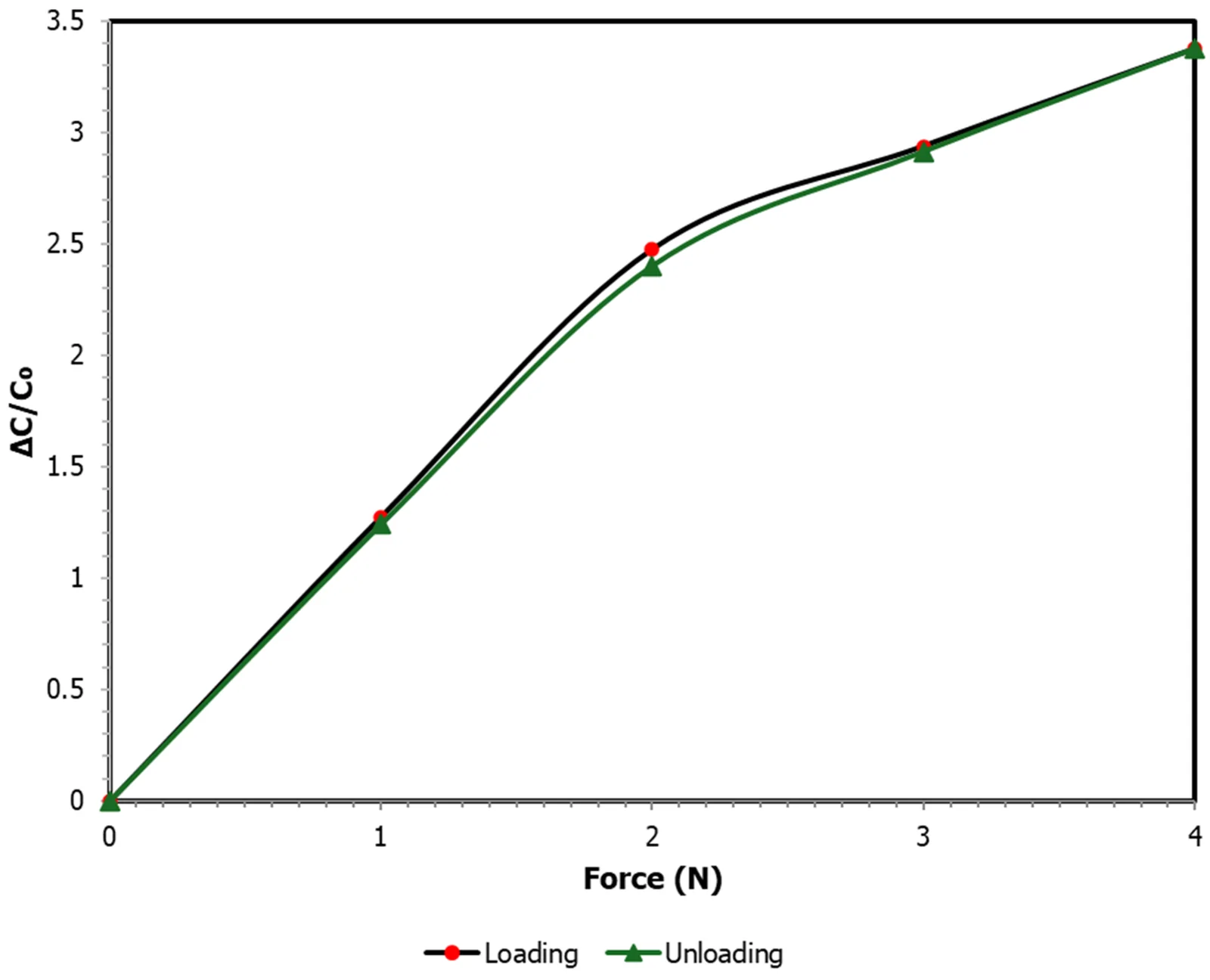
A plot of the capacitance versus force of the palm sensor when loaded up to 4 N and unloaded, showing the hysteresis.

The complete force loading/unloading cycle from 0 to 4 N and back to 0 N was performed on a single sensing element at 1 N increments. Figure 7 illustrates the hysteresis characteristics. The maximum hysteresis error of only 1.9% FSO occurs around 1.5 N. This minimal hysteresis indicates that the elastic recovery of the folded sponge dielectric is highly reversible, with negligible energy dissipation, despite the foam material’s complex internal structure.

The curves converge perfectly at both the 0 N and 4 N endpoints, confirming complete elastic recovery and the absence of permanent deformation after loading cycles. The consistently small deviation between loading and unloading paths (typically < 0.1 in ΔC/C_0_) throughout the measurement range demonstrates the sensor’s suitability for dynamic force measurement applications where accurate force tracking during both grip tightening and release is essential for object manipulation tasks in robotic hands.

## 4. Hardware and Software Implementation

The complete data acquisition pipeline integrates sensors via an ESP32-S3 (Espressif Systems, Shanghai, China), which is connected to the computer for data storage and processing. The sensors are sampled at 66.7 Hz. The detailed schematic of the hardware is presented in the Supplementary Information. The system demonstrates that sparsely placed sensors combined with appropriate signal conditioning achieve robust tactile perception without requiring complex distributed computation.

A semi-elastic textile glove with minimal stretch characteristics was selected as the mounting substrate for all sensors, providing sufficient compliance for comfortable wear while limiting elastic deformation that could introduce measurement artefacts during hand motion. Each sensor was adjusted so that the upper electrode segment aligns with the center of the proximal interphalangeal joint (PIP) and the lower electrode segment aligns with the center of the metacarpophalangeal joint (MCP).

**Figure 8:**
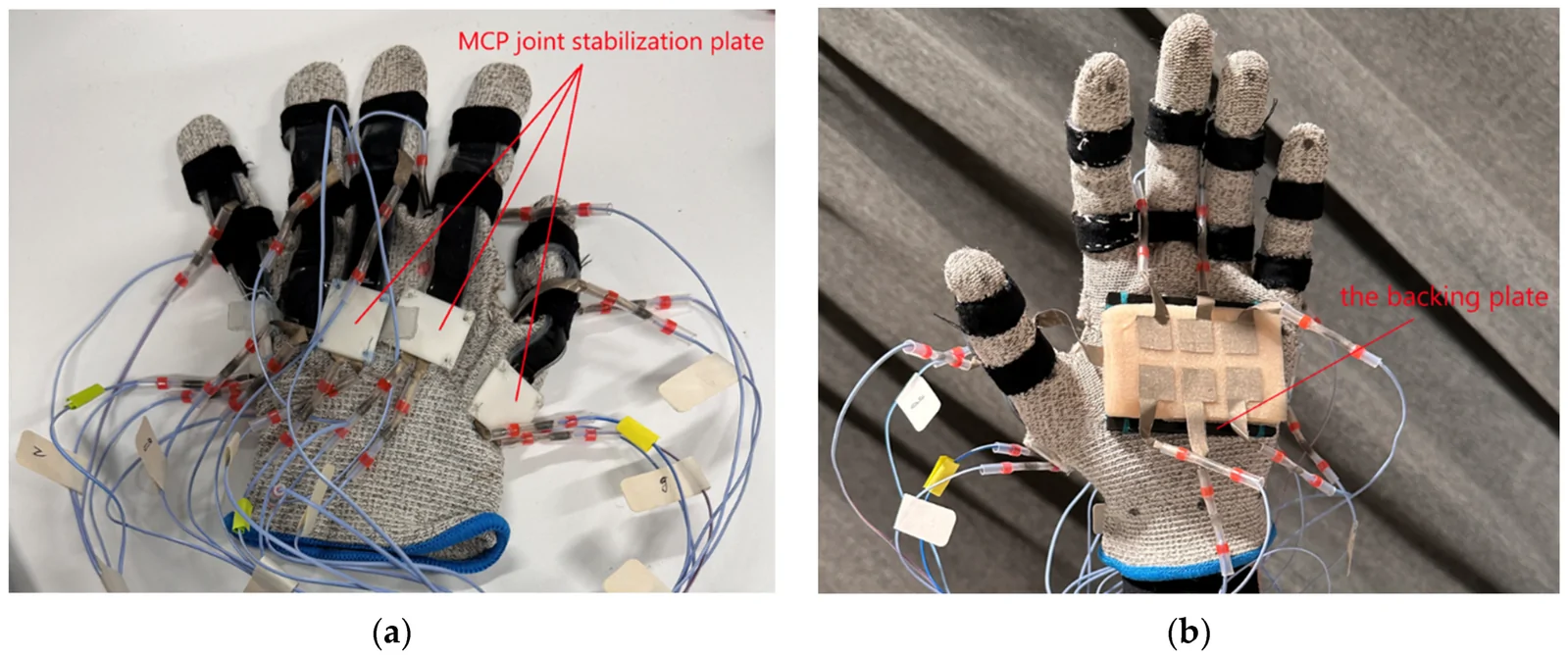
(**a**) Stretch sensor installation showing elastic tape fixation, stitching points, and PLA stabilization plates; (**b**) Palm pressure sensor array mounted on rigid PLA backing plate.

The pure Ecoflex side lateral tabs extending from the sensor core wrap around the finger, secured with unidirectional elastic tape (circular elasticity only, no longitudinal elasticity), allowing unrestricted longitudinal finger movement. Sutures were passed through the Ecoflex flaps at multiple points along the sensor edge to anchor it to the glove fabric, while the finger is in a neutral position to avoid introducing pre-strain. Small 3D-printed PLA plates (15 mm × 12 mm, with four corner mounting holes) cover the MCP joint area. This plate presses against the pure Ecoflex region beneath the lower electrode segment, initially secured with double-sided tape, then permanently sutured to the glove base through the corner holes. This multi-point fixation strategy ensures that sensor strain during finger flexion originates from joint angle changes rather than sensor slippage. See Figure 8a.

Palm pressure sensors mounted on a rigid PLA backing plate (65 mm × 50 mm, 2 mm thick) have multiple functions: preventing localized sensor deformation under eccentric loads, limiting palm compliance to restrict natural hand soft tissue deformation (simulating robotic hand’s palm rigidity), and isolating pressure sensors from glove material deformation. This ensures that measured contact force distributions reflect object geometry rather than tissue compliance, thereby enhancing the transferability of recognition models trained on human grasping data to robotic applications. The backing plate is secured to the palm of the glove with double-sided tape, then stitched through the corner mounting holes using a cross-stitch pattern. The complete six-element pressure sensor assembly is affixed to the backing plate exposed surface with double-sided tape, with six sensing elements aligned to anatomical contact areas. See Figure 8b.

## 5. Object Shape Recognition Using Multi-Layer Perceptron (MLP)

This section addresses object recognition using MLP, aiming to distinguish four fundamental geometric primitives, i.e., cuboids, spheres, cylinders, and cones, across multiple sizes to demonstrate that a compact, strategically configured capacitive tactile sensing system can support robust shape recognition for robotic hand applications. A standardized dataset is constructed through the fabrication of representative objects and the execution of a controlled, repeatable grasping protocol, while a systematic feature selection strategy is applied to retain the most discriminative capacitive sensing channels, thereby reducing system complexity and improving generalization. An MLP classifier is adopted for its suitability for low-dimensional structured data and computational efficiency, and the overall recognition framework integrates data preprocessing, network architecture design, and structured dataset partitioning to ensure reliable and generalizable performance.

An MLP was selected as the method for shape recognition due to its simplicity. Compared to Convolutional Neural Networks (CNNs), MLPs have a simpler architecture without convolution or pooling operations, making them easier to implement and better suited to small, low-dimensional datasets, such as the 11-sensor feature vector discussed later. MLPs are more scalable than Support Vector Machines (SVMs), which suffer from a quadratic computational complexity during kernel matrix computation. Relative to k-Nearest Neighbors (KNN), MLPs offer significantly higher inference speed by requiring only a single forward pass through the network rather than distance calculations against all training samples. Finally, MLPs can learn complex nonlinear feature hierarchies via multiple hidden layers, whereas Random Forests (RFs) are limited to axis-aligned splits and thus struggle with diagonal decision boundaries and feature interactions.

The fully connected feedforward architecture of MLPs enables automatic feature learning from raw sensor data while maintaining compact model size (KB-scale parameters) and sub-millisecond inference latency, both critical for deployment on resource-constrained robotic controllers operating in real-time control loops.

It is important to emphasize that machine learning in this framework serves as an evaluation and integration tool, rather than the primary contribution of the work. The classifier quantifies how effectively different tactile sensing configurations encode object geometry. The emphasis of this section is therefore on sensor placement, modality selection, and system simplification, supported by learning-based fusion to enable objective comparison.

### 5.1. Experimental Objects

Twelve objects were fabricated from polylactic acid (PLA) using 3D printing, representing four fundamental geometric shapes across three size scales. The shape categories, cuboid, sphere, cylinder, and cone, were selected to span a range of geometric complexities while representing primitives commonly encountered in robotic manipulation scenarios.

**Figure 9:**
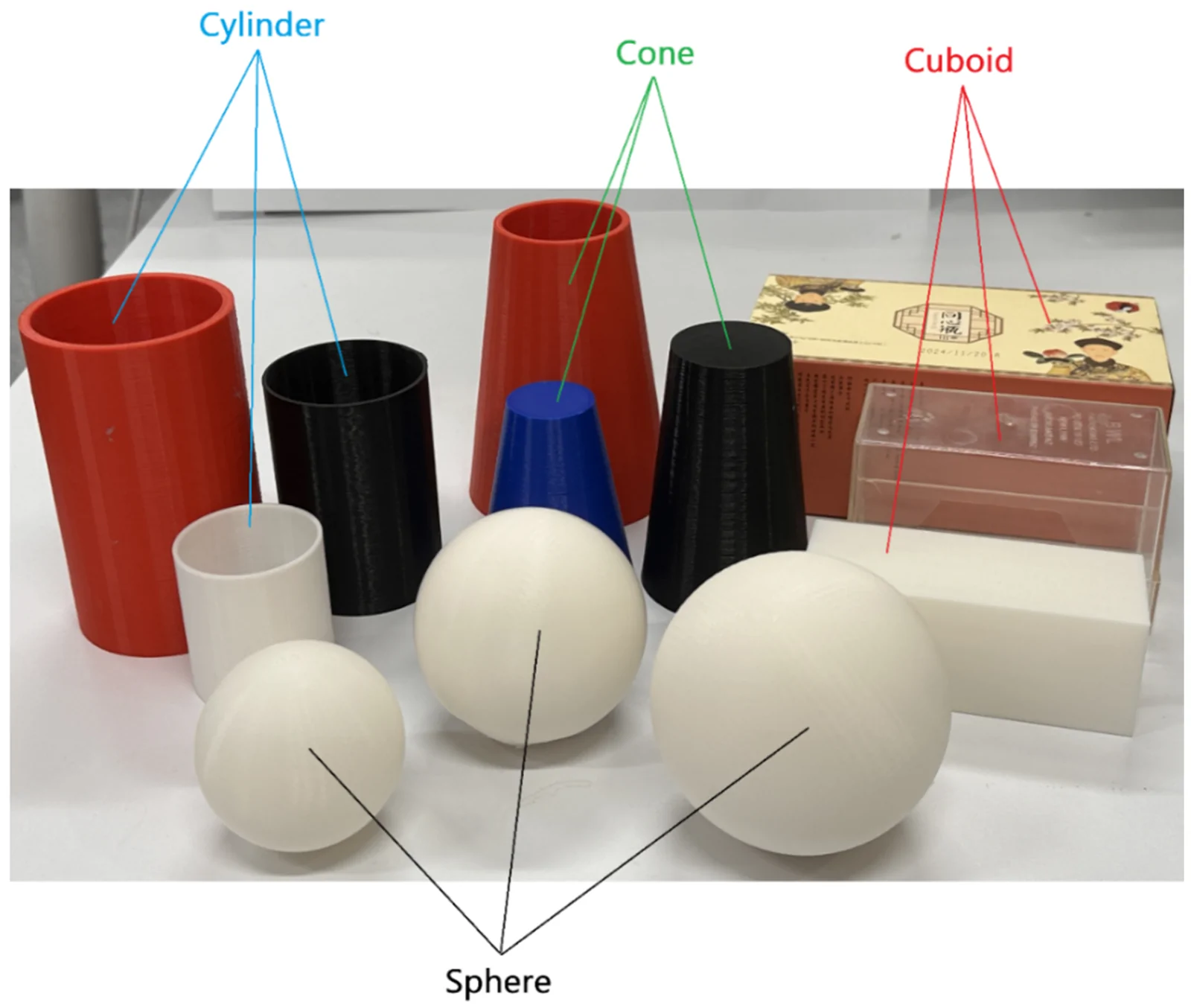
Photo of the experimental objects to be grasped by the hand.

Table 3 details the physical specifications of all experimental objects. Object dimensions were constrained to the natural hand-grasping range (50–100 mm characteristic dimension), ensuring comfortable power grasp execution during the 100-trial repetition protocol while remaining representative of real-world object manipulation. Figure 9 shows a photo of all 12 experimental objects.

### 5.2. Data Acquisition Protocol

Data acquisition followed the quasi-static grasping protocol, designed to capture stable contact configurations representative of robotic hand manipulation. For each of the 12 experimental objects, the protocol consisted of five steps: object presentation, robotic grasp emulation (grasping with all five fingers wrapping around the object), stabilization period (2–3 s for force equilibration), data capture trigger (recording 16-dimensional sensor vector), and then release, with a repetition of 100 trials per object.

**Figure 10:**
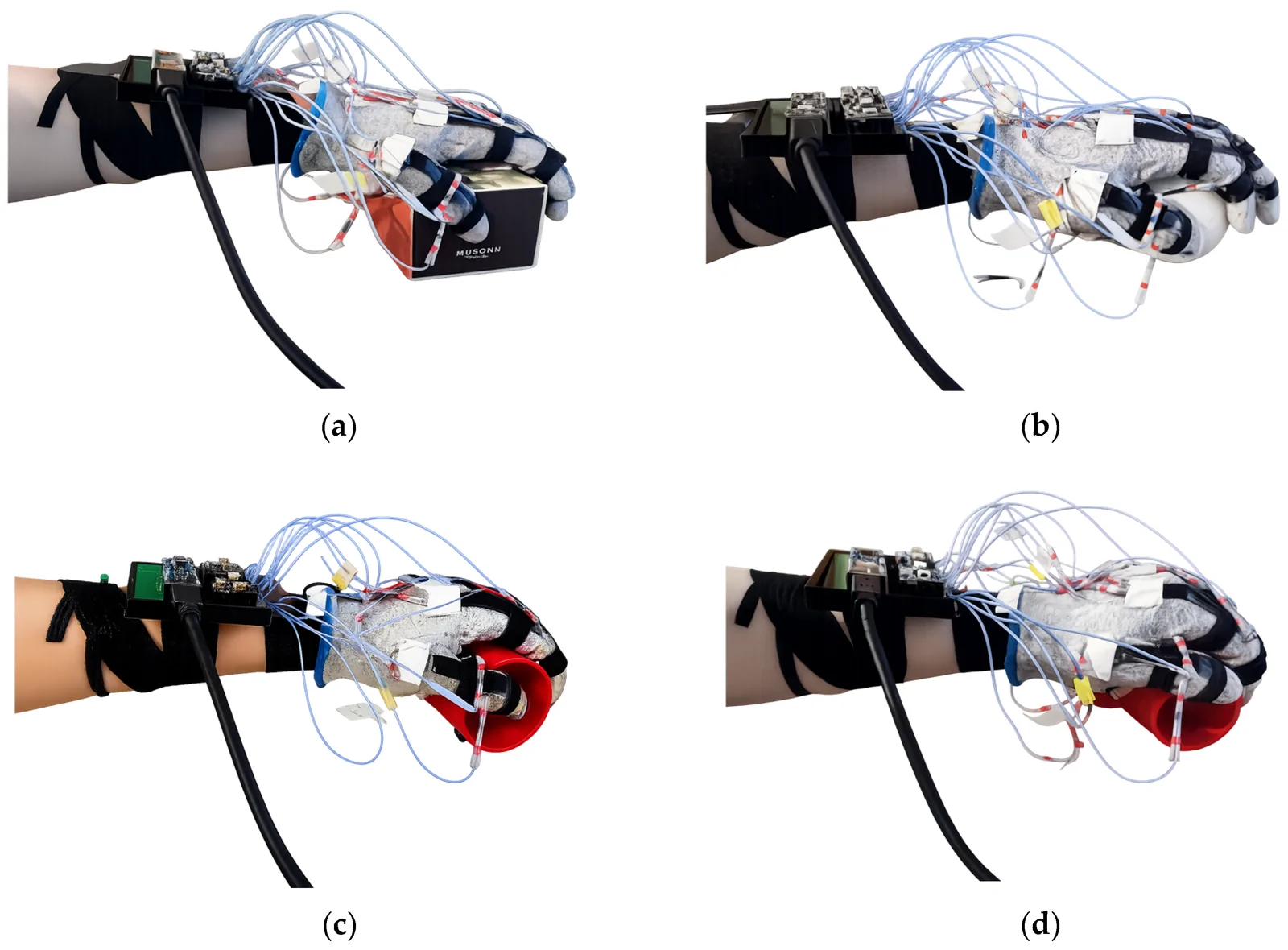
Grasping configurations of the hand on (**a**) cuboid, (**b**) sphere, (**c**) cylinder, (**d**) cone. (Note: AI was used to remove the background in the images to enhance visibility.)

Figure 10 shows the grasping configurations for each of the four shape categories. The hand approaches the object from the top to mimic how a robotic hand would grasp. The rigid PLA palm backing plate mechanically constrained the natural compliance of human palm tissue while approximating the rigid palm structure characteristic of robotic hands. This ensures that measured force distributions reflect the object geometry rather than hand-tissue compliance.

The final dataset comprises 1200 measurements, structured hierarchically into four shape categories × 300 samples each (i.e., 100 samples per shape variant: small, medium, and large). This balanced structure ensures equal representation of all shape classes during training. The dataset was designed for size-invariant recognition, in which instances of the same shape across different sizes are treated as a single class rather than as distinct categories. During both training and testing, samples from all three size levels are intermingled within each shape category, requiring the classifier to extract geometric features that generalize across sizes rather than memorizing size-specific cues.

### 5.3. Sensor Channel Selection

**Figure 11:**
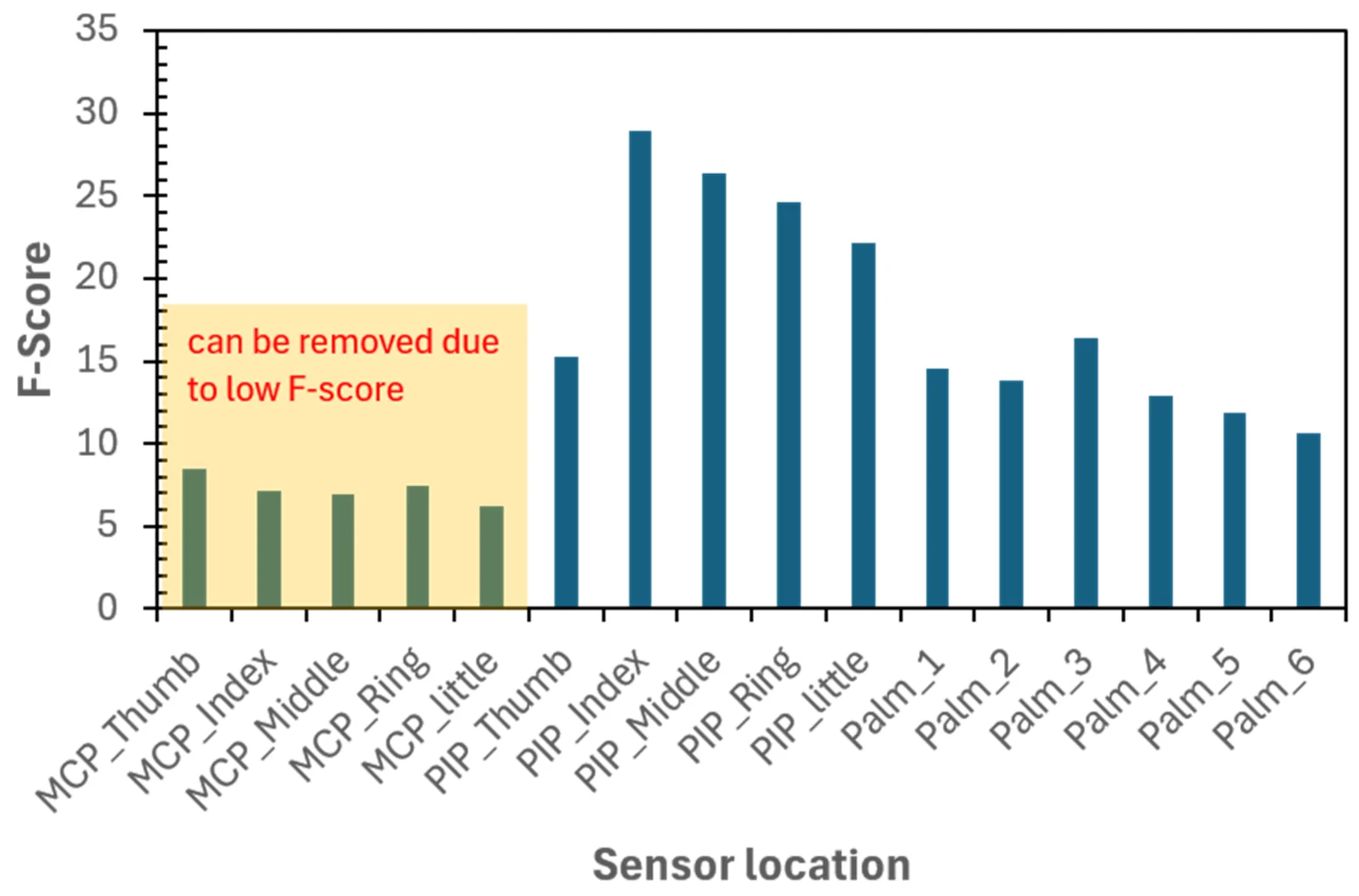
ANOVA F-scores for all sensor channels.

The initial sensor setup provided 16-dimensional feature vectors comprising: 10 finger-joint sensors (i.e., five MCP and five PIP joints) and six palm pressure sensors. Preliminary analysis using ANOVA F-test statistics revealed significant variation in sensor discriminative capacity across the four shape classes. The resulting F-scores for all data from the 16 channels are summarized in Figure 11.

The five MCP joint sensors exhibited relatively low discriminative performance, with F-scores ranging from 6.23 to 8.45, substantially lower than those of the PIP sensors (15.23–28.91) and the palm pressure sensors (10.67–16.43). Since MCP joints primarily reflect gross hand aperture and user-specific anatomical variability rather than geometry-dependent contact patterns, their signals introduced high within-class variance and offered limited discriminatory value. Based on this analysis, all five MCP sensors were excluded from the feature set, reducing the input dimensionality from 16 to 11 channels. The final retained configuration comprises five PIP joint sensors (i.e., one per finger, including thumb) and six palm pressure sensors (arranged in a 2 × 3 spatial array), preserving all geometry-sensitive measurements while eliminating noisy signals.

### 5.4. Data Preprocessing

Raw sensor measurements were pre-processed using two-stage normalization to ensure numerical stability during MLP training:

1. Stage 1—Z-score standardization

For each sensor channel j, every measurement $x_{ij}$ in sample i was standardized according to:

(2) $${x_{ij}}^{std}=\frac{\left(x_{ij}-\mu_{j}\right)}{\left(\sigma_{j}+\varepsilon \right)}$$

where $\mu_{j}$ and $\sigma_{j}$ donate the mean and standard deviation computed across all training samples for that sensor channel j. A small constant, $\varepsilon =10^{-8}$, was added to prevent division by zero. This step centers each sensor’s distribution at zero mean with unit variance.

2. Stage 2—Min–max scaling to [−1, 1]

After standardization, each channel was further transformed using bounded min–max scaling:

(3) $${x_{ij}}^{norm}=2\times \frac{{x_{ij}}^{std}-x_{min,j}}{x_{max,j}-x_{min,j}+\varepsilon }-1$$

where $x_{min,j}$ and $x_{max,j}$ are the minimum and maximum values in the standardized data. This maps all values to the symmetric interval [−1, 1], providing optimal input conditions for LeakyReLU activation functions. (Note: LeakyReLU is used as the activation function in hidden layers to improve learning stability and avoid dead neurons.)

### 5.5. MLP Architecture

**Figure 12:**
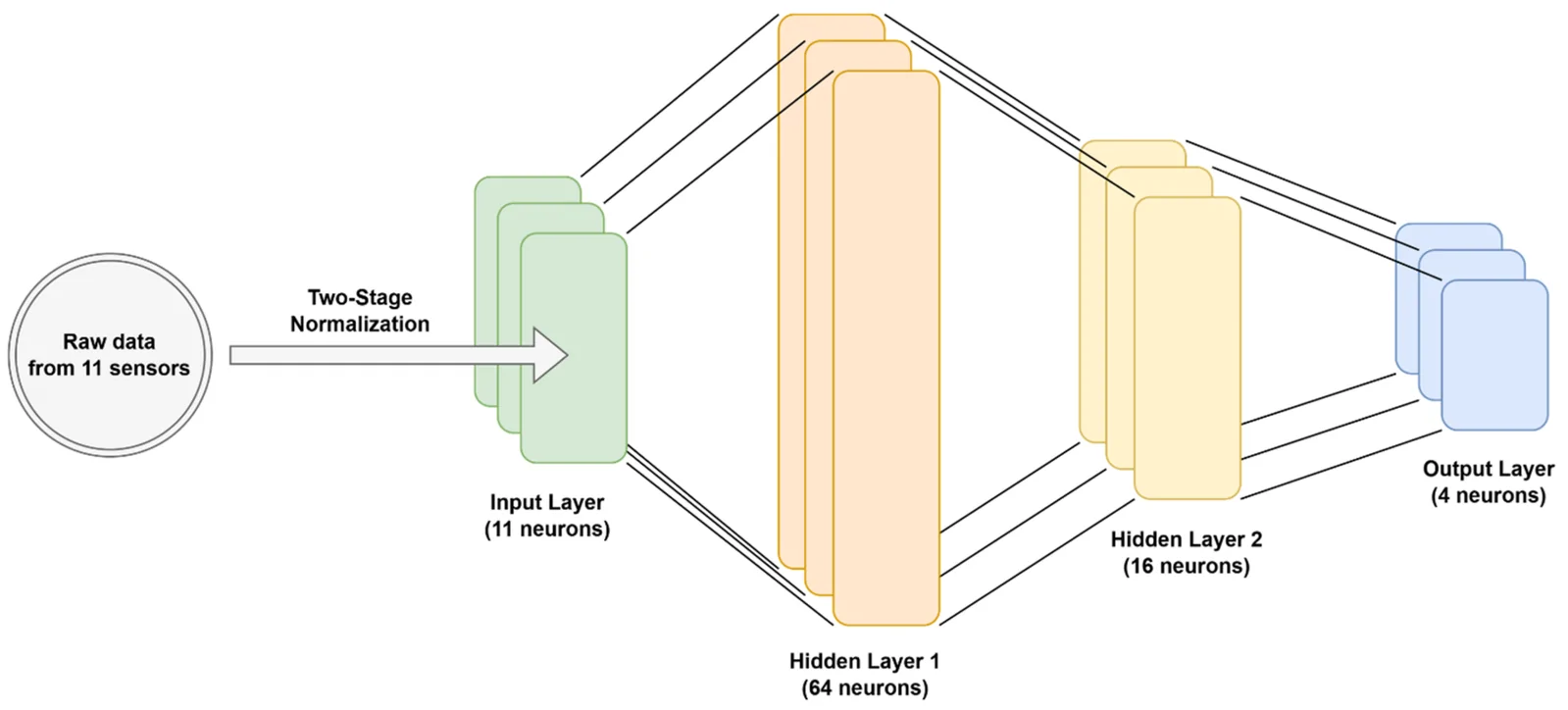
Architecture of the MLP.

The implemented MLP architecture follows an 11 → 64 → 16 → 4 hourglass pattern (expansion–compression–recognition) that balances representational capacity with compact parameterization. This design philosophy prioritizes efficient learning while maintaining the compact model size and fast inference requirements established in the Recognition Method Section. Figure 12 illustrates the complete network structure.

The network architecture is configured according to empirically established scaling heuristics for shallow neural networks. The first hidden layer expands the 11-dimensional input to 64 neurons, corresponding to an approximate sixfold increase in dimensionality. Such an expansion is commonly used for low-dimensional inputs to provide sufficient representational capacity to capture nonlinear structure in raw sensor measurements. The second hidden layer reduces the intermediate representation to 16 neurons, forming a bottleneck whose size is proportional to the number of target classes. This bottleneck enforces the extraction of salient geometric features while implicitly regularizing the model and reducing the risk of overfitting. The final layer maps the learned features to four shape categories using a softmax activation function.

The network was trained using categorical cross-entropy loss optimized with the Adam algorithm. The learning rate was set to α = 5 × 10^−4^ with standard momentum parameters β_1_ = 0.9 and β_2_ = 0.999. Hidden layers employ LeakyReLU activation functions with a negative slope (α = 0.3) to prevent gradient vanishing, while the output layer uses softmax for a multi-class probability distribution. Weights were initialized using He-Initialization to ensure proper gradient flow during early training. The model was trained for 200 epochs with a batch size of 4, yielding 240 parameter updates per epoch. The conservative learning rate ensures stable gradient descent without oscillation during convergence.

### 5.6. Training Configuration

After normalization, the full dataset of 1200 samples was partitioned using stratified random sampling. Specifically, 240 samples per class were allocated to the training set (i.e., 960 samples total), and the remaining 60 samples per class were used for testing (i.e., 240 samples total), resulting in an 80/20 train–test split. Four MLP models with identical architecture were trained using different sensor configurations: 16-channel configuration: all sensors (five MCP + five PIP + six palm); 11-channel configuration: selected sensors (five PIP + six palm); 5-channel configuration: PIP stretch sensors only; 6-channel configuration: palm pressure sensors only.

The MLP model was trained for 200 epochs with a batch size of 4, resulting in 240 parameter updates per epoch. Test performance was evaluated at the end of each epoch to monitor training progression and assess generalization.

**Figure 13:**
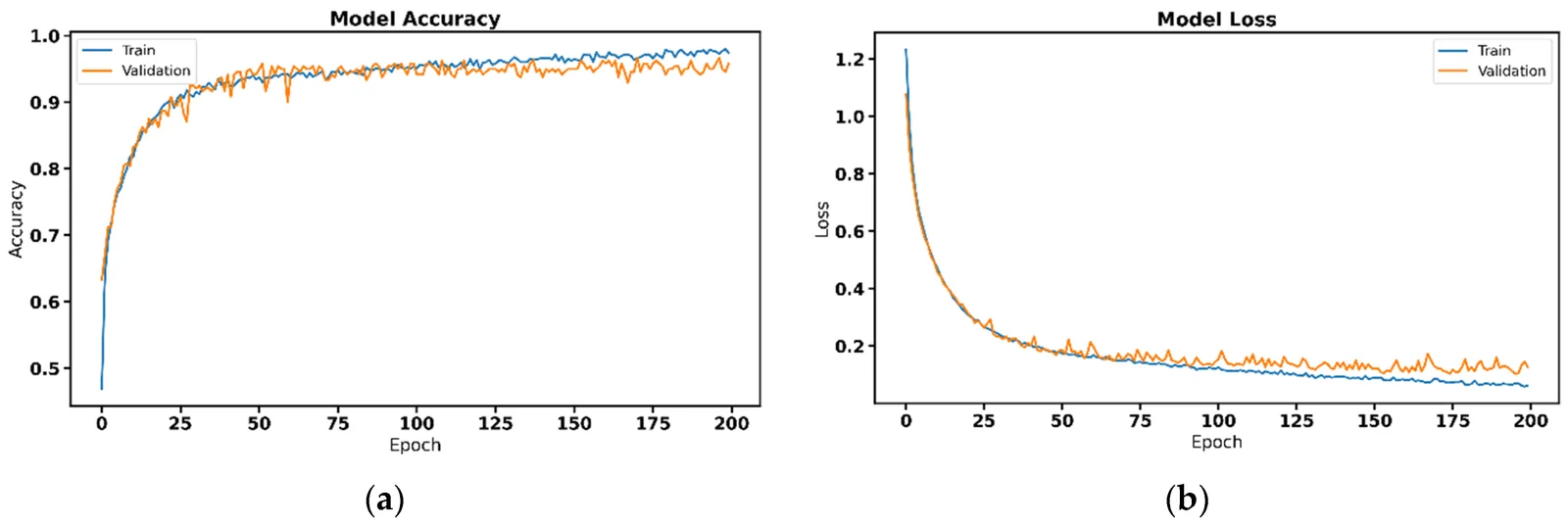
(**a**) Accuracy curves and (**b**) loss curves over 200 epochs.

The MLP training exhibited rapid initial convergence followed by stable plateau behavior, as illustrated in Figure 13a. Training accuracy increased sharply from the random-chance baseline of 25% to approximately 90% within the first 25 epochs, demonstrating effective gradient-based learning of discriminative sensor patterns. By around epoch 50, both training and test accuracy exceeded 95%, with training accuracy continuing to improve gradually toward 98%, while test accuracy stabilized around 95–97%.

The loss curves shown in Figure 13b exhibit the same convergence pattern, with the categorical cross-entropy loss decreasing rapidly from approximately 1.3 to below 0.3 within the first 30 epochs. Throughout training, the test loss closely tracked the training loss, suggesting limited overfitting despite the relatively small dataset (12 predefined rigid objects across four geometric primitive classes). The modest train–test gap (i.e., around 3% in accuracy and approximately 0.05 in loss) indicates that the bottleneck-style architecture provides implicit regularization, reducing the likelihood of sample memorization.

The stable plateau observed after epoch 50 suggests that the network had effectively converged, with later epochs contributing only marginal improvement. This rapid convergence validates the effectiveness of the preprocessing pipeline (i.e., z-score standardization followed by min–max scaling) and the conservative learning rate (5 × 10^−4^), which together enable stable gradient descent without oscillation or overshooting.

## 6. Recognition Performance Comparison

**Figure 14:**
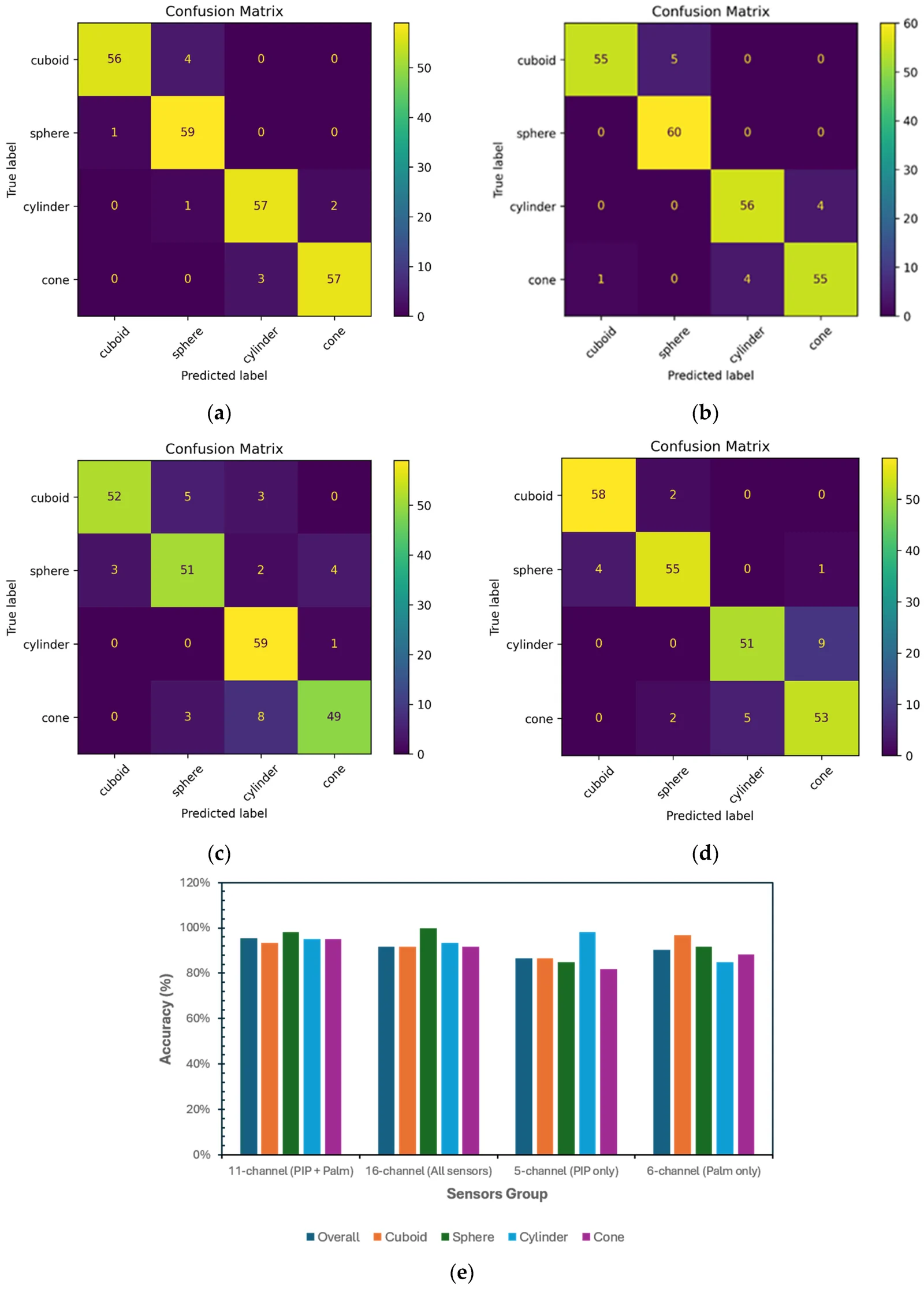
Confusion matrices for four sensor configurations: (**a**) 11-channel (PIP + Palm), (**b**) 16-channel (All sensors), (**c**) 5-channel (PIP only), (**d**) 6-channel (Palm only), (**e**) Comparative recognition accuracy across different sensor configurations.

The study evaluated shape recognition performance using four different sensor configurations—11-channel (PIP + palm sensors), 16-channel (all sensors), 5-channel (PIP only), and 6-channel (palm only)—and the results are summarized in Figure 14.

The 11-channel configuration delivered the strongest overall performance, achieving 95.4% accuracy, 3.7% higher than the 16-channel setup. Although it may seem surprising that using fewer sensors produced better results, the findings indicate that several MCP sensors contributed little useful information and instead introduced noise. This supports the well-known concept that adding irrelevant features can reduce model performance rather than improve it.

While the 16-channel configuration achieved perfect recognition of spheres, its performance on cuboids, cylinders, and cones was lower, resulting in reduced overall accuracy. This suggests that simply increasing the number of sensors does not necessarily lead to better generalization.

The 5-channel PIP-only configuration achieved 86.7% accuracy and performed particularly well in recognizing cylinders. However, it struggled with spheres and cones, suggesting that finger flexion information alone is insufficient to capture certain shape characteristics.

The 6-channel palm-only configuration achieved 90.4% accuracy and excelled at recognizing cuboids. However, it performed worse on cylinders, suggesting that pressure information alone cannot reliably distinguish all object shapes.

By combining both sensor types, the 11-channel configuration effectively leveraged their complementary strengths. PIP sensors provided information about finger flexion and curvature, while palm sensors captured force distribution across the hand. This integration resulted in consistently high recognition rates above 93% for all shape classes and improved overall accuracy by 5 percentage points compared with the best single-modality configuration.

Overall, the results demonstrate that the 11-channel sensor arrangement offers the best balance between recognition accuracy, number of sensors, and computational efficiency, making it the most effective configuration for four-class shape recognition, and MCP information is not necessary for object shape recognition.

## 7. Conclusions and Future Work

This study introduces a complete object recognition framework for grasp-based tactile perception using a limited number of capacitive sensors. By combining biomechanically informed sparsely placed sensors and machine learning recognition, the work establishes a practical approach suitable for robotic hand applications. Experimental results across several sensor configurations show that MCP joint sensors offer limited discriminative power and are therefore redundant. Removing these low-value channels reduces the number of sensors from 16 to 11 and improves accuracy by 3.7 percentage points, demonstrating that redundant channels can hinder generalization. The recognition model, an MLP with an 11 → 64 → 16 → 4 architecture, effectively extracts features while maintaining computational efficiency suitable for embedded systems. Remaining errors primarily stem from geometric ambiguities, such as similarities between cylinders and cones or edge-contact cases for cuboids and spheres. Ablation studies confirm that both proximal interphalangeal stretch sensors and palm pressure sensors are essential, validating the 11-channel design as a sufficient configuration. It must be acknowledged that only MLP was evaluated, as the objective is not to perform an extensive benchmarking study across classifiers. MLP serves as part of the overall recognition framework, and the reported results reflect the combined sensing–learning system rather than the sensing hardware in isolation.

Beyond performance, the sensing framework offers practical engineering benefits: fewer channels reduce hardware complexity and computation while preserving accuracy. The work highlights that strategically placed sensors outperform dense sensor layouts under resource constraints.

Algorithmically, the framework currently relies on static grasp data. Future work should incorporate temporal models and expand datasets across users, hand geometries, grasp styles, and object types, including deformable and articulated objects, to enhance generalization. Overall, the study demonstrates that a reduced sensing configuration, achieved by removing the MCP sensors, is sufficient for recognition under the experimental conditions examined in this work. Future work should evaluate additional object categories, grasp strategies, users, robotic hand geometries, and alternative machine learning approaches to better assess the generality of the findings.

## Figures and Tables

**Table 1:** Joint-angle and palmar-force characteristics for different object geometries.

Object	Hand Gesture	Palm Force Distribution
Cylindrical	Progressive MCP-PIP flexion forming a smooth arc with moderate, uniform angles	Elongated, longitudinal force distribution with gradual gradients
Conical	Systematic joint angle variation; greater flexion near the apex than base	Asymmetric, wedge-shaped force gradient with higher pressure at narrow end
Cuboidal	More extended fingers with specific angles aligned to flat faces	Rectangular pressure map with sharp boundaries; force at edges and vertices
Spherical	Large, uniform MCP-PIP flexion forming a dome-shaped hand	Centralized, circular, radially symmetric force pattern

**Table 2:** Stretch sensor dimensional specifications.

Parameter	Standard Variant (Index, Middle, Ring)	Compact Variant (Thumb, Little Finger)	Notes
Total Length	80 mm	60 mm	Full sensor, including all layers
Bottom Electrode (Layer 2)	70 mm × 10 mm	50 mm × 10 mm	Continuous CB/Ecoflex
Top Electrode—PIP Segment	25 mm × 10 mm	15 mm × 10 mm	PIP Segment
Top Electrode—MCP Segment	35 mm × 10 mm	25 mm × 10 mm	MCP Segment
Inter-electrode Gap	10 mm	10 mm	Pure Ecoflex isolation
Core Width	10 mm	10 mm	Central electrode region
Lateral Tab Extensions	Variable (trimmed to fit)	Variable (trimmed to fit)	Mounting tabs

**Table 3:** Physical dimensions of experimental objects (all PLA material; Ø indicates diameter, h indicates height).

Shape	Size	Dimensions (mm)
Cuboid	Small	60 × 40 × 80
	Medium	75 × 55 × 100
	Large	90 × 70 × 120
Sphere	Small	Ø60
	Medium	Ø80
	Large	Ø100
Cylinder	Small	Ø50 × 80 (h)
	Medium	Ø65 × 100 (h)
	Large	Ø80 × 120 (h)
Cone	Small	Ø60 × 80 (h)
	Medium	Ø80 × 100 (h)
	Large	Ø100 × 120 (h)

## Data Availability

Unavailable due to privacy or ethical restrictions.

## Supplementary Materials

The following supporting information can be downloaded at: https://www.mdpi.com/article/10.3390/s26175583/s1, Figure S1. Manufacturing process of finger stretch sensors for (a) layer 1, (b) layer 2, (c) layer 3, (d) layer 4, (e) layer 5; Figure S2. The materials and components used in the construction of a palm pressure sensor: (a) plan view, (b) angled view, (c) final appearance of the fully assembled palm pressure sensor (i) front, (ii) back, and (iii) cross-section; Figure S3: Complete system schematic showing FDC1004 capacitive sensing frontend, TCA9548A I^2^C multiplexer, and ESP32-S3 microcontroller.
