# Supplementary material for: Object Shape Recognition Using Sparse Soft Capacitive Tactile Sensors for Robotic Hands
Source: Sensors (Basel). 2026 Sep 2;26(17):5583. doi: 10.3390/s26175583 (PMC13568368; doi:10.3390/s26175583)
Supplement: Supplementary file 1 [file sensors-26-05583-s001.zip › sensors-4445706-supplementary.pdf]

Supplementary Information

Stretch Sensor

Materials

Carbon black (CB) powder (Vulcan® XC72R purchased from Fuel Cell Store, Texas, USA) serves as the conductive material for flexible capacitor electrodes. Ecoflex™ 00-30 (Smooth-On Inc.) serves as both the electrode matrix (when mixed with CB) and the dielectric layer material (pure form), providing exceptional elongation properties (up to 900% strain), Shore hardness of 00-30 for high compliance, low Young's modulus (~125 kPa) for minimal mechanical interference, chemical compatibility with carbon black, and stable relative permittivity $\varepsilon_r \approx 2.8$ across the measurement frequency range (25 kHz).

CB:Ecoflex mass ratio of 1:10 (9.1% by mass, slightly above percolation threshold) achieves optimal balance between electrical conductivity (enabling low-impedance electrodes), mechanical flexibility (maintaining electrode compliance during stretching), and fabrication consistency (ensuring reproducible mixing and uniform electrode properties).

Fabrication Process

The detailed fabrication steps are as follows in Figure S1:

Step 1: Mold Preparation: An acrylic base plate that provides a rigid foundation and flat working surface.

Step 2: Layer 1 - Bottom Encapsulation (Pure Ecoflex), as shown in Figure S1 (a). Pure Ecoflex™ 00-30 (without carbon black) was prepared by mixing Part A and Part B in an equal mass ratio (1:1) according to manufacturer specifications.

Step 3: (Figure S1(b)): Layer 2 - Bottom Electrode (CB/Ecoflex) with Conductive Fabric; the conductive CB/Ecoflex composite was prepared by first mixing Ecoflex™ 00-30 Part A and Part B in 1:1 mass ratio.

Step 4: Layer 3 - Middle Insulation (Pure Ecoflex); after curing the second layer, another layer of pure Ecoflex™ 00-30 was cast over the bottom electrode. See Figure S1 (c). The pure Ecoflex was degassed and poured to completely cover the bottom electrode.

Step 5: Layer 4 - Top Electrode (Segmented CB/Ecoflex) with Conductive Fabrics; the fourth layer consists of two separate CB/Ecoflex electrode segments, formed using the layer 4 segmented stencil. See Figure S1 (d). CB/Ecoflex composite material was poured into the upper and lower electrode cavities defined by the stencil, then leveled using an acrylic scraper.

Step 6: Layer 5 - Top Encapsulation (Pure Ecoflex); a final layer of pure Ecoflex™ 00-30 was cast over the entire fourth layer structure. See Figure S1 (e).

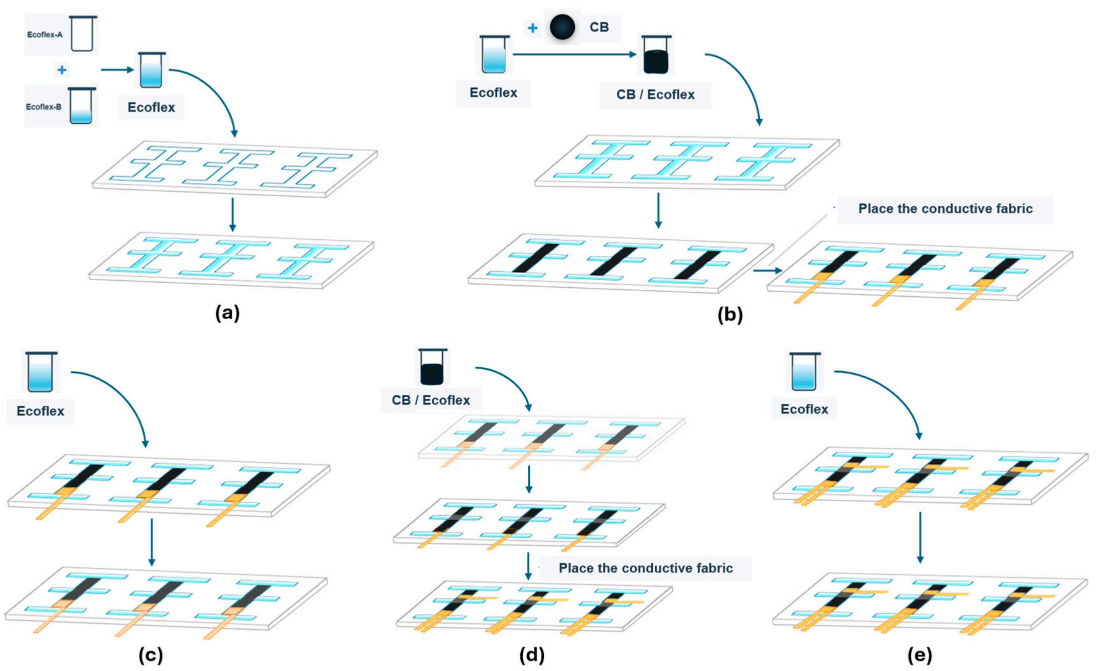

Figure S1. Manufacturing process of finger stretch-sensors for (a) layer 1, (b) layer 2, (c) layer 3, (d) layer 4, (e) layer 5

Palm Pressure Sensor

Materials

Palm pressure sensors employ conductive fabric electrodes (Ni/Cu radiation-shielding fabric, < 1 Ω/sq) that provide electrical conductivity, flexibility for conformal contact, and durability under repeated compression. The compressible dielectric consists of synthetic sponge (FYOUGA Skin Film) with an open-cell structure, folded to achieve a final thickness of ~5mm when no force is applied, providing increased baseline thickness for a larger capacitance change range, progressive compression characteristics for improved sensitivity, and mechanical resilience that prevents permanent deformation.

Fabrication Process

The components required for constructing a palm pressure sensor as shown in Figure S2 (a). The detailed fabrication steps are as follows:

Step 1 - Bottom Electrode Preparation: A continuous sheet of conductive fabric was cut to dimensions 60 mm × 35 mm using precision scissors to serve as the common ground electrode. An extension (5 mm × 55 mm) was incorporated at one edge to provide a reliable electrical connection to the measurement circuitry.

Step 2 - Sponge Dielectric Preparation: A compressible, open-cell sponge sheet was selected as the dielectric layer. The sponge was folded into several layers to achieve a final uncompressed dimension of 65 mm x 40 mm and a thickness of approximately 5 mm.

Step 3 - Top Electrode Array Fabrication: Six individual conductive fabric sensing elements were carefully cut to 12 mm × 12 mm dimensions using precision scissors to ensure dimensional consistency. Each electrode patch included a 6 mm × 45 mm extension tail for subsequent wire connection.

Step 4 - Layer Assembly: The three layers were assembled in sequence on a flat work surface. First, the bottom electrode layer was positioned flat on the workbench surface, serving as the common ground plane for the capacitive sensor array. Next, the pre-folded sponge medium layer was aligned centrally on the bottom electrode. Finally, the six top electrode patches were arranged in a 2×3 array layout with a 5 mm pitch in both directions, fully covering the primary palm contact area during grasping.

Step 5 - Structural Fixation: Transparent adhesive tape strips were applied along the perimeter of the assembly. First, four tape strips were applied along the outer edge to securely bond the sponge dielectric layer to the bottom electrode substrate. Additional tape segments were then added between adjacent electrode plates to prevent lateral displacement during deformation. Finally, directional tape bonding was performed in the central sponge region to ensure uniform compression characteristics while maintaining electrical isolation. Figure S2(b) shows the final appearance of the fully assembled palm pressure sensor.

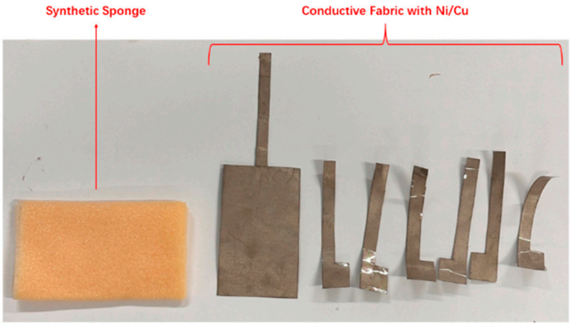

(a)

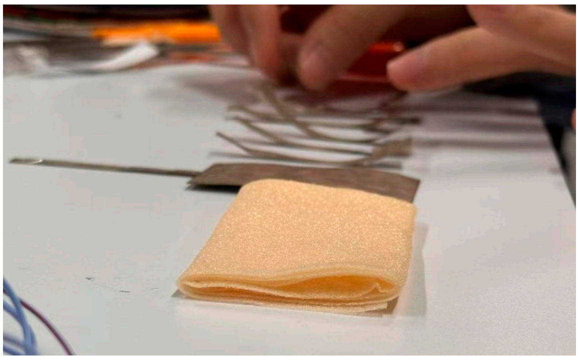

(b)

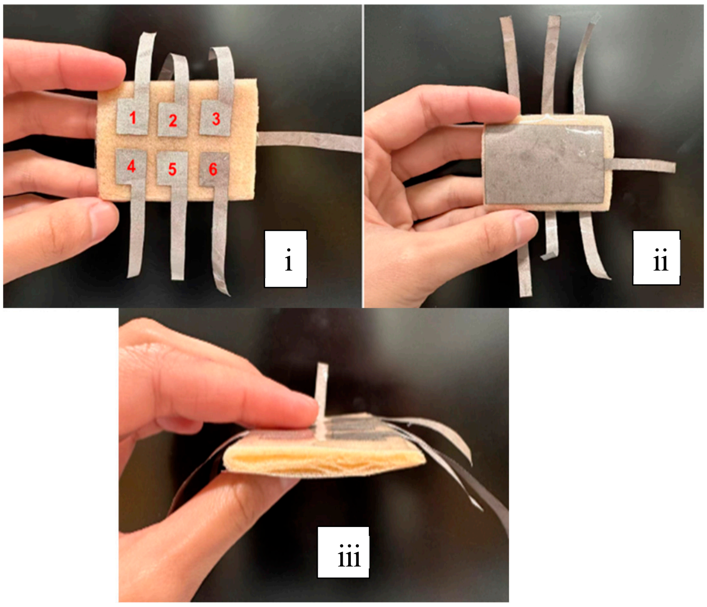

(c)

Figure S2. The materials and components used in the construction of a palm pressure sensor; (a) plan view (b) angled view, (c) Final appearance of the fully assembled palm pressure sensor (i) front, (ii) back side, and (iii) cross-section of the palm pressure sensor

Interface Electronics

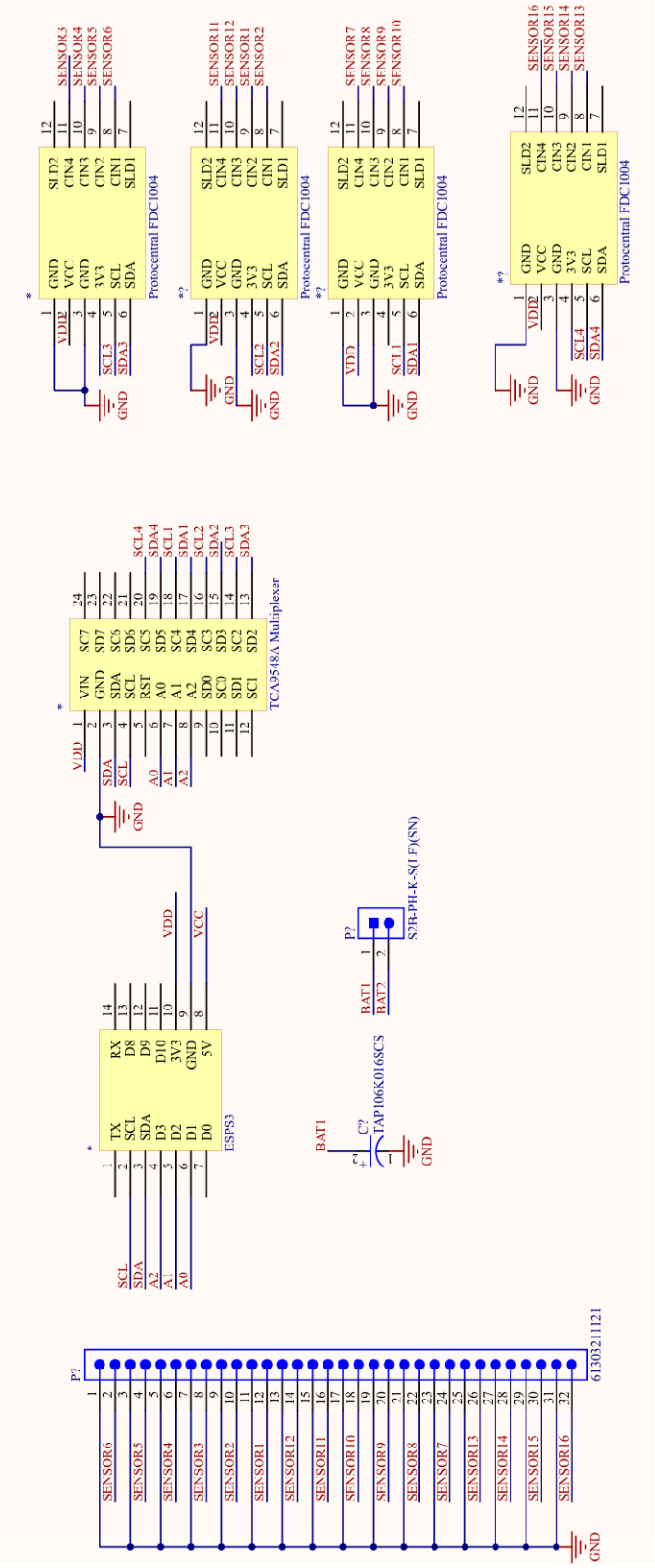

Figure S3: Complete system schematic showing FDC1004 capacitive sensing frontend, TCA9548A I²C multiplexer, and ESP32-S3 microcontroller
